# Treatment-related pneumonitis after thoracic radiotherapy/chemoradiotherapy combined with anti-PD-1 monoclonal antibodies in advanced esophageal squamous cell carcinoma

**DOI:** 10.1007/s00066-024-02199-6

**Published:** 2024-01-24

**Authors:** Xiaoyan Lv, Yajing Wu, Qihui Li, Chen Zheng, Qiang Lin, Qingsong Pang, Min Zhao, Jiandong Zhang, Jun Wang

**Affiliations:** 1 Department of Radiation Oncology, the Fourth Hospital of Hebei Medical University, Hebei Clinical Research Center for Radiation Oncology, Shijiazhuang, China; 2 Department of Oncology, North China Petroleum Bureau General Hospital, Hebei Medical University, Renqiu, China; 3 Department of Radiation Oncology, Tianjin Medical University Cancer Institute & Hospital, Tianjin, China; 4 Department of Oncology, the First Hospital of Hebei Medical University, Shijiazhuang, China; 5 Department of Oncology, The First Affiliated Hospital of Shandong First Medical University and Shandong Province Qianfoshan Hospital, Shandong Lung Cancer Institute, Jinan, China; 6 Department of Oncology, Shandong First Medical University, Jinan, China; 7 Department of Radiation Oncology, the Fourth Hospital of Hebei Medical University, 050011 Shijiazhuang, China

**Keywords:** Esophageal squamous cell carcinoma, Thoracic radiotherapy, Anti-PD‑1 monoclonal antibodies, Pneumonitis, Dosimetric parameters

## Abstract

**Purpose:**

This study aims to evaluate the risk factors of treatment-related pneumonitis (TRP) following thoracic radiotherapy/chemoradiotherapy combined with anti-PD‑1 monoclonal antibodies (mAbs) in patients with advanced esophageal squamous cell carcinoma (ESCC).

**Methods:**

We retrospectively reviewed 97 patients with advanced ESCC who were treated with thoracic radiotherapy/chemoradiotherapy combined with anti-PD‑1 mAbs. Among them, 56 patients received concurrent radiotherapy with anti-PD‑1 mAbs and 41 patients received sequential radiotherapy with anti-PD‑1 mAbs. The median prescribed planning target volume (PTV) dose was 59.4 Gy (range from 50.4 to 66 Gy, 1.8–2.2 Gy/fraction). Clinical characteristics, the percentage of lung volume receiving more than 5–50 Gy in increments of 5 Gy (V_5_–V_50_, respectively) and the mean lung dose (MLD) were analyzed as potential risk factors for TRP.

**Results:**

46.4% (45/97), 20.6% (20/97), 20.6% (20/97), 4.1% (4/97), and 1.0% (1/97) of the patients developed any grade of TRP, grade 1 TRP, grade 2 TRP, grade 3 TRP, and fatal (grade 5) TRP, respectively. Anti-PD‑1 mAbs administered concurrently with radiotherapy, V_5_, V_10_, V_15_, V_25_, V_30_, V_35_, V_40_ and MLD were associated with the occurrence of grade 2 or higher TRP. Concurrent therapy (*P* = 0.010, OR = 3.990) and V_5_ (*P* = 0.001, OR = 1.126) were independent risk factors for grade 2 or higher TRP. According to the receiver operating characteristic (ROC) curve analysis, the optimal V_5_ threshold for predicting grade 2 or higher TRP was 55.7%.

**Conclusion:**

The combination of thoracic radiotherapy/chemoradiotherapy with anti-PD‑1 mAbs displayed a tolerable pulmonary safety profile. Although the incidence of TRP was high, grade 1–2 TRP accounted for the majority. Anti-PD‑1 mAbs administered concurrently with radiotherapy and the lung V_5_ were significantly associated with the occurrence of grade 2 or higher TRP. Therefore, it seems safer to control V_5_ below 55% in clinical, especially for the high-risk populations receiving concurrent therapy.

**Supplementary Information:**

The online version of this article (10.1007/s00066-024-02199-6) contains supplementary material, which is available to authorized users.

## Introduction

Esophageal cancer is the seventh most common malignancy in terms of morbidity and the sixth most common cause of cancer-related death worldwide [1]. The KETNOTE-181 study [2], ATTRACTION-03 study [3], and ESCORT study [4] had established the efficacy and safety of immune checkpoint inhibitors (ICIs) in patients with advanced or metastatic esophageal squamous cell carcinoma (ESCC) after first-line standard therapy. The addition of ICIs to chemotherapy has also demonstrated superior efficacy to chemotherapy in patients with untreated, advanced or metastatic esophageal cancer in the KEYNOTE-590 study [5] and the ESCORT-1st study [6]. The management of advanced esophageal cancer, especially the combination of thoracic radiotherapy and ICIs, is still evolving. There are three ongoing phase 3 randomized controlled studies of ICIs concurrently with chemoradiotherapy for locally advanced unresectable esophageal cancer (KEYNOTE-975 study [7], NCT04426955 study, and RATIONALE-311 study [8]). However, the synergistic anti-tumor effects of RT and ICIs can also lead to overlapping toxicity profiles [9]. Hence, pulmonary toxicities following combined therapy have attracted increasing attention.

Previous studies on anti-PD-1/PD-L1 monoclonal antibodies (mAbs) have reported varying incidence of ICI-related pneumonitis, ranging from 0 to 10% [10]. However, no significant correlation was found between the dose of ICIs and the occurrence of ICIs-related pneumonitis [11]. In comparison, the occurrence of pneumonitis related to anti-PD‑1 mAbs was slightly higher compared with anti-PD-L1 mAbs (3.6% vs. 1.3%), especially in grade 3–4 pneumonitis (1.1% vs. 0.4%) [12]. A subgroup analysis of the KEYNOTE 001 trial found that patients with non-small cell lung cancer (NSCLC) who had previously received thoracic radiotherapy were more likely to have any-grade pulmonary toxicity, especially pneumonitis and respiratory failure [13]. However, there is no relevant evidence for esophageal cancer thus far. More real-world data are needed to further evaluate pulmonary toxicity in combined therapy using ICIs. And it is also urgent to adjust lung irradiation constraint thresholds based on dosimetric parameters in the era of ICIs. Although other analogous studies on lung cancer have been published, there is no consensus on the relationship between treatment-related pneumonitis (TRP) and dosimetric parameters. For instance, Jabbour et al. and Voong et al. noticed that there was no significant correlation between ICI-related pneumonitis and RT parameters in NSCLC patients who previously received thoracic radiotherapy [14, 15]. The PACIFIC trial revealed a significant incidence of any-grade pneumonitis/radiation pneumonitis (33.9%) in NSCLC patients receiving concurrent chemoradiotherapy followed by durvalumab consolidation therapy, and the occurrence of pneumonitis was not related to previous RT dose [16]. Although the PACIFIC trial included 25.2% of Asians in the experimental group, current data does not include a secondary analysis of pulmonary toxicity in this population. In contrast, two Japanese studies found that V_20_ was a risk factor for grade ≥ 2 pneumonitis in Asian patients with NSCLC who received concurrent chemoradiotherapy followed by Durvalumab [17, 18]. In fact, data on lung cancer cannot be extrapolated to esophageal cancer due to tumor heterogeneity. To address this lack of specific data, we conducted a retrospective study to further explore the pulmonary toxicity of combination strategies in the Asian population with ESCC.

There were several specific aims for this study, Firstly, to assess the pulmonary safety of combining thoracic radiotherapy with anti-PD‑1 mAbs for advanced ESCC and compare the pulmonary toxicity between different combination modalities (concurrent vs sequential). Secondly, to explore the risk factors of TRP after combined therapy. Finally, to evaluate dosimetric parameters as predictors of symptomatic TRP and explore the constraints of lung irradiation in the context of ICIs.

## Materials and methods

### Patients

Patients who met the following criteria were eligible for this study: confirmed diagnosis of ESCC, received thoracic radiotherapy for primary tumor, regional lymph node metastasis and/or anastomotic/esophageal recurrence, administration of anti-PD‑1 mAbs either concurrently or after thoracic radiotherapy, and periodic imaging reviews to enable grading of TRP.

Exclusion criteria were as follows: concurrent presence of other malignancies, active or previous history of autoimmune disease, evidence of uncontrolled or ongoing illness or infections, history of thoracic re-radiotherapy, history of interstitial lung disease treated with steroids or active noninfectious pneumonitis, insufficient follow-up imaging data, and incomplete radiotherapy dosimetry data.

We reviewed the medical, radiological, and radiation therapy records of all the patients treated at our institution between May 2019 and March 2021. Ninety-seven patients met the inclusion criteria. They all received intensity modulated radiation therapy (IMRT). Of these, 21 had upper thoracic esophageal cancer, 59 had middle thoracic esophageal cancer, and 17 had lower thoracic esophageal cancer; 75 patients had unresectable, locally advanced/metastatic ESCC that had not been previously treated, whereas 22 had recurrent/metastatic esophageal cancer after radical esophagectomy; 28 of 41 patients with stage IV ESCC and 13 of 56 patients with stage III ESCC received concurrent ICI with RT. We collected the following demographic and clinical data (summarized in Table 1), age (range, 38–84 years; median, 64 years), gender, smoking history, Eastern Cooperative Oncology Group (ECOG) performance status score, baseline lung function, dosimetric parameters, prior therapy, combination regimen of radiotherapy and immunotherapy, and type of anti-PD‑1 mAb.

**Table 1:** Characteristics of 97 patients

Characteristics	Classifications	No. (%) (*n* = 97)
Sex	Female	31 (32.0)
	Male	66 (68.0)
Median age, years (range)	64 (38–84)	–
ECOG	0–1	82 (84.5)
	2	15 (15.5)
History of smoking	No	47 (48.5)
	Yes	50 (51.5)
History of chronic pulmonary diseases	No	75 (77.3)
	Yes	22 (22.7)
Initial cancer stage	III	56 (57.7)
	IV	41 (42.3)
Radiotherapy combined with ICI	Concurrent	41 (42.2)
	Sequential	56 (57.8)
MLD, Gy (range)	10.9 (6.7–15.7)	
Median V_5_, % (range)	50.3 (27.9–73.6)	
Median V_20_, % (range)	21.3 (12.7–29.9)	
ICI agents	Camrelizumab	69 (71.1)
	Pembrolizumab	8 (8.2)
	Sintilimab	8 (8.2)
	Tislelizumab	1 (1.0)
	Toripalimab	11 (11.3)
Prior esophagectomy	No	76 (78.4)
	Yes	21 (21.6)
Concurrent chemotherapy	None	14 (14.4)
	Paclitaxel and cisplatin	38 (39.2)
	Paclitaxel and nedaplatin	6 (6.2)
	Paclitaxel and carboplatin	4 (4.1)
	Fluoropyrimidine and cisplatin	8 (8.2)
	Fluoropyrimidine and oxaliplatin	4 (4.1)
	Irinotecan and cisplatin	6 (6.2)
	Docetaxel and cisplatin	5 (5.2)
	Tegafur	8 (8.2)
	Paclitaxel	3 (3.1)
	Capecitabine	1 (1.0)

*ECOG* Eastern Cooperative Oncology Group performance status score. History of chronic pulmonary diseases, including chronic obstructive pulmonary disease, emphysema, interstitial lung disease, chronic bronchitis, asthma, pulmonary tuberculosis, and pulmonary hypoplasia

### Assessment and dosimetric parameters

The observation and evaluation of TRP began when patients received anti-PD‑1 mAbs and ended at the time of death or at the end of follow-up. Prior to the initiation of ICIs, patients were required to undergo a baseline lung CT examination (baseline CT). Radiographic examinations were performed every 1–3 months after ICI initiation. The diagnosis and grading of TRP were confirmed by two experienced radiation oncologists based on clinical symptoms, chest imaging, and evidence of medication in the medical records. TRP was assessed by the Common Terminology Criteria for Adverse Events Version 5.0 (CTCAE V5.0) and recorded according to highest grade observed during follow-up. We defined sequential immunotherapy as the interval between the end of thoracic radiotherapy and the administration of anti PD‑1 monoclonal antibodies exceeding 7 days.

All patients underwent contrast-enhanced CT scans, with a 3 mm slice interval, and the CT images were transmitted to the Pinnacle8c planning system for 3D reconstruction and analysis of radiation dose distribution and lung doses. The dose-volume histogram (DVH) parameters were extracted from the treatment planning system, including planning target volume (PTV), mean lung dose (MLD) and lung V_5–50_ in 5 Gy increments. The dose prescribed to cover 95% of PTV was1.8–2.2 Gy per fraction, five fractions per week, for a total of 50.4–66 Gy.

### Statistical analysis

SPSS 23.0 software was utilized for statistical analysis. Non-normally distributed data were analyzed using the Mann-Whitney rank-sum test for two independent samples. Means or medians were calculated for continuous variables and compared using the t‑test. Multivariate analysis included variables with statistical significance or showed a trend (*P* < 0.1) in the univariate analysis and binary logistic forward stepwise regression was used. Analysis of DVH parameter using the receiver operating characteristics (ROC) curve was also performed to select the cut-off value to predict grade ≥ 2 TRP. *P* < 0.05 was considered statistically significant.

## Results

The follow-up continued until September 30, 2021. The median follow-up time was 12.3 months (IQR 9.3–20.6). Thirty (31%) patients had died at the point of data cutoff, while 17 patients were still receiving anti-PD‑1 mAbs and 80 patients had stopped.

### TRP

In this study, 46.4% (45/97), 20.6% (20/97), 20.6% (20/97), 4.1% (4/97), and 1.0% (1/97) of the patients developed any grade of TRP, grade 1 TRP, grade 2 TRP, grade 3 TRP, and fatal (grade 5) TRP, respectively. In the anti-PD‑1 mAbs administered concurrently with radiotherapy/chemoradiotherapy group, 48.4% (20/41), 12.2% (5/41), 29.3% (12/41), 12.3% (3/41) of the patients developed any grade of TRP, grade 1 TRP, grade 2 TRP, grade 3 TRP, respectively. In the anti-PD‑1 mAbs administered sequentially with radiotherapy/chemoradiotherapy group, 44.6% (25/56), 26.8% (15/56), 14.3% (8/56), 1.8% (1/56), 1.8% (1/56) of the patients developed any grade of TRP, grade 1 TRP, grade 2 TRP, grade 3 TRP, and grade 5 TRP, respectively. There were no significant differences in the dosimetric parameters between two groups (Table 2). The CT images of 5 patients with grade 3 or higher TRP are available in Supplement Fig. 1.

**Table 2:** The DVH parameters of 97 patients

Parameters	All groups (Mean ± SD)	Different groups		
		Concurrent	Sequential	*P* value
V_5_ (%)	49.2 ± 8.8	48.8 ± 9.3	49.5 ± 8.5	0.688
V_10_ (%)	35.9 ± 5.5	35.8 ± 5.7	36.0 ± 5.5	0.909
V_15_ (%)	27.4 ± 4.6	27.7 ± 4.7	27.2 ± 4.5	0.569
V_20_ (%)	21.3 ± 3.9	21.5 ± 4.2	20.7 ± 3.5	0.312
V_25_ (%)	15.4 ± 3.5	15.8 ± 3.7	15.1 ± 3.4	0.320
V_30_ (%)	10.8 ± 3.7	11.2 ± 3.6	10.6 ± 3.8	0.382
V_35_ (%)	7.4 ± 3.4	7.6 ± 3.3	7.3 ± 3.5	0.574
V_40_ (%)	4.8 ± 2.8	4.9 ± 2.6	4.7 ± 2.9	0.730
V_45_ (%)	3.0 ± 2.2	3.0 ± 2.0	3.0 ± 2.3	0.932
V_50_ (%)	1.7 ± 1.5	1.8 ± 1.6	1.7 ± 1.5	0.834
MLD (cGy)	1093.7 ± 196.5	1098.6 ± 207.6	1093.8 ± 189.8	0.996

*DVH* dose-volume histogram, *SD* standard deviation, *MLD* mean lung dose, *V*_*5*_*–V*_*60*_ percentage of lung volume receiving more than 5–50 Gy

**Table 3:** The associations between clinical, treatment, dosimetric variables and G2+ treatment-related pneumonitis

Risk factors	G2+ *vs.* G 0–1 pneumonitis		
	G2+ (*n* = 25)	G 0–1 (*n* = 72)	*P* value
*Sex*			
Male	15	51	0.317
Female	10	21	
Median age, years (range)	65 (48–84)	64 (38–83)	0.518
*ECOG*			
0–1	20	62	0.467
≥ 2	5	10	
*Smoking history*			
No	13	24	0.680
Yes	12	38	
*History of chronic lung diseases*			
No	18	57	0.461
Yes	7	15	
*Radiotherapy combined with ICI*			
Concurrent	15	26	0.037
Sequential	10	46	
*Concurrent chemotherapy*			
No	14	28	0.137
Yes	11	44	
*PTV dose*			
< 60 Gy	11	38	0.449
≥ 60 Gy	14	34	
*Dose fractionation*			
1.8 Gy	5	10	0.463
2 Gy	15	53	
> 2 Gy	5	9	
V_5_, % (Mean ± SD)	54.0 ± 7.7	47.6 ± 8.6	0.001
V_10_, % (Mean ± SD)	38.4 ± 4.5	35.0 ± 5.6	0.006
V_15_, % (Mean ± SD)	29.2 ± 4.3	26.8 ± 4.6	0.026
V_20_, % (Mean ± SD)	22.3 ± 3.6	20.6 ± 3.9	0.052
V_25_, % (Mean ± SD)	16.7 ± 3.2	15.0 ± 3.6	0.042
V_30_, % (Mean ± SD)	12.4 ± 3.6	10.3 ± 3.7	0.011
V_35_, % (Mean ± SD)	8.8 ± 3.3	6.9 ± 3.3	0.009
V_40_, % (Mean ± SD)	5.8 ± 3.0	4.5 ± 2.6	0.048
V_45_, % (Mean ± SD)	3.6 ± 2.4	2.8 ± 2.1	0.085
V_50_, % (Mean ± SD)	2.1 ± 1.8	1.6 ± 1.4	0.166
MLD, Gy (Mean ± SD)	1196.7 ± 179.8	1058.0 ± 1 90.3	0.001

*ECOG* Eastern Cooperative Oncology Group performance status score, *SD* standard deviation, *MLD* mean lung dose, *V*_*5*_*–V*_*60*_ percentage of lung volume receiving more than 5–50 Gy

The median onset time of TRP was 16 weeks (2.6 weeks–65.9 weeks) after the initiation of anti- PD‑1 mAbs and 19.6 weeks (0.3 weeks–89.0 weeks) after the initiation of thoracic radiotherapy. Overall, 12 of 97 patients (12.4%) who received anti-PD‑1 mAbs were treated with corticosteroids for pneumonitis, including 7 with grade 2 TRP and 5 with grade 3 or higher TRP. Of the 12 patients, 7 (58.3%) were not rechallenged with ICIs after treatment with corticosteroids. Another 5 patients with grade 2 TRP had their pneumonitis resolved to ≤ grade 1 and discontinued corticosteroids by the time of rechallenge. Grade 3 pneumonitis relapsed in 1 of 5 patient leading to ICIs discontinuation. Detailed individual information and treatment of 5 patients with G3 + pneumonitis is shown in Supplement Table 1. Notably, one patient (1.8%) in the sequential group developed refractory pneumonitis 153 days after the initiation of radiotherapy (54 days after the initiation of ICIs). The patient received a total of 2 cycles of Pembrolizumab. Despite receiving corticosteroid treatment and mechanical ventilation support, the patient died of pneumonitis two months later.

According to CTCAE V5.0, G2 pneumonitis is defined as symptomatic and requires medical intervention. Therefore, we dichotomized pneumonitis to G0–1 vs G2 or higher to analyze the risk factors for TRP. Univariate analysis showed that anti-PD‑1 mAbs administered concurrently with radiotherapy, V_5_, V_10_, V_15_, V_25_, V_30_, V_35_, V_40_ and MLD were associated with the occurrence of G2 or higher TRP (Table 3). Variables with *P* < 0.1 in univariate analysis (concurrent therapy, V_5_, V_10_, V_15_, V_20_, V_25_, V_30_, V_35_, V_40_, V_45_ and MLD) were included in the multivariate analysis, which showed that concurrent therapy (*P* = 0.010, OR = 3.990) and V_5_ (*P* = 0.001, OR = 1.126) were independent risk factors for grade ≥ 2 TRP (Table 4).

**Table 4:** Binary logistic regression of the association between treatment, dosimetric variables and G2+treatment-related pneumonitis

	B	Standard error	Wald value	Degrees of freedom	*P* value	Exp(B)	95%CI
V_5 (%)_	0.119	0.036	10.635	1	0.001	1.126	1.049–1.210
Concurrent therapy	1.384	0.537	6.641	1	0.010	3.990	1.393–11.430

*V*_*5*_ percentage of lung volume receiving more than 5 Gy

**Fig. 1:**
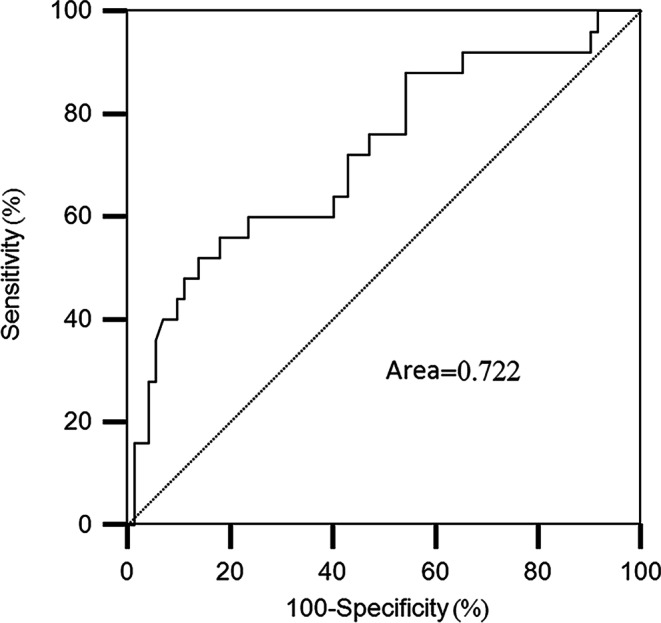
ROC curve and the associated area for V_5_ as a predictor for grade 2 or higher treatment-related pneumonitis

The results of the ROC curve analysis are shown in Fig. 1. The area under the ROC curve was 0.722 (95%CI: 0.600–0.843), and the optimal threshold for V_5_ was 55.7%. The incidence of grade 2 or higher (G2+) TRP was 56.5% (13/23) in patients with V_5_ values higher than the determined threshold (55.7%), whereas it was 16.2% (12/74) in patients with V_5_ values lower than the determined threshold.

**Fig. 2:**
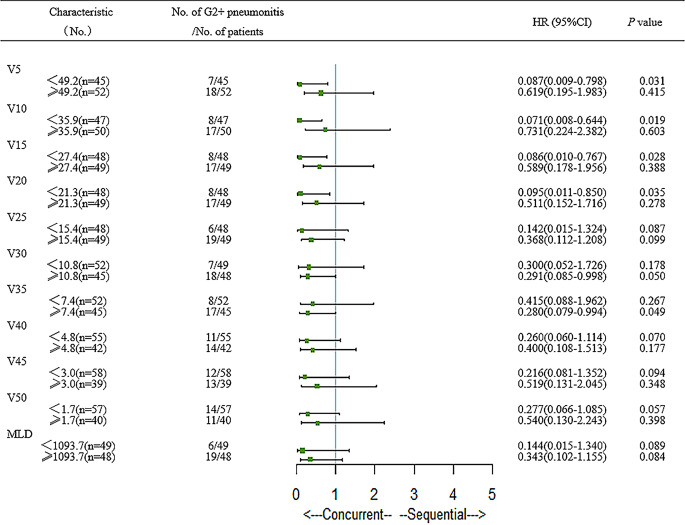
Forest plots show factors associated with G2+ treatment-related pneumonitis in entire cohort

We then performed subgroup analysis between ICIs administered concurrently with radiotherapy group and ICIs administered sequentially with radiotherapy group. In the subgroup analysis of G2+ TRP, patients with a gender of male, history of smoking, history of chronic lung diseases, V_5_ < 49.2%, V_10_ < 35.9%, V_15_ < 27.4%, V_20_ < 21.3%, V_35_ ≥ 7.4% had significantly increased the risk of the occurrence of G2+ TRP in the concurrent group (Fig. 2).

## Discussion

The success of chemoimmunotherapy as second-line treatment for advanced or metastatic ESCC, and even as the first-line standard treatment, has prompted research into the possibility of adding radiotherapy to triple therapy. The synergistic anti-tumor mechanism of radiotherapy and ICIs has served as the basis for combining radiotherapy with standard chemoimmunotherapy. In previous clinical studies on chemoimmunotherapy for advanced or metastatic ESCC, the reported incidence of pneumonitis was 7%, and the incidence of grade 5 pneumonitis was between 0 and 1%.

The patients included in the ATTRACTION-03 study and ESCORT study both had ESCC, among which 70 and 64% had received radiotherapy, respectively [3, 4]. In the ATTRACTION-03 study, the incidence of pneumonitis was 9.2 and 5.6% in the nivolumab group and chemotherapy group who received radiotherapy, compared with 7.0 and 7.6% in those who did not receive radiotherapy, suggesting that radiotherapy may increase the risk of pneumonitis after ICIs [3]. However, there is no secondary analysis in patients who have previously thoracic radiotherapy currently. The incidence of pneumonitis was 7.8% (6/77) in the Japanese subgroup treated with pembrolizumab and 6.5% (4/62) in the Chinese subgroup [20] in KEYNOTE-181 [19]. However, no study further analyzed squamous cell carcinoma patients who had previously received thoracic radiotherapy. These summative data reveal that there is lack of comprehensive analysis of pulmonary toxicity after thoracic radiotherapy combined with ICIs in Asian patients with ESCC.

After conventionally fractionated radiotherapy, the first radiological manifestation of radiation pneumonitis is expected to occur within 6 months, and approximately 15% of patients develop pneumonitis within 2 to 3 months [21, 22]. The median onset time of immune pneumonitis was 2.8 months after the initiation of ICIs [23]. However, for patients receiving thoracic radiotherapy combined with anti-PD‑1 mAbs, it is usually difficult to clearly distinguish between radiation pneumonitis and immune pneumonitis in clinical practice. Therefore, the endpoint of our study was TRP. A subgroup analysis of a meta-analysis that involved six clinical trials showed that the incidence of grade 3 or higher pneumonitis was 1.9% in patients with locally advanced esophageal cancer who were received concurrent and sequential CRT/RT and ICI [24]. However, the use of this combination therapy in trials may not reflect the real-world data. The results of this study corroborated the tolerable pulmonary safety of combined therapy. Although the incidence of pneumonitis was much higher than that reported in previous studies, it was mainly low grade. A secondary analysis of the phase I trial KEYNOTE-001 [13] showed that although thoracic radiotherapy increased the probability of pulmonary toxicity (12.5% vs. 1.4%), the incidence of high-grade pulmonary toxicity was similar (4.2% vs. 1.4%).

The primary novelty of this study pertains to include the patients who received concurrent radiotherapy/chemoradiotherapy plus anti-PD‑1 mAbs, which is very rare even compared to other analogous studies of lung cancer. In this study, 28 of 41 patients with stage IV ESCC received concurrent therapy. Chemoimmunotherapy is the standard treatment for stage IV ESCC. For this population, palliative RT could alleviate dysphagia. Additionally, some retrospective studies had shown that delivering RT to primary tumor could might improve survival [25, 26]. There were 13 of 56 patients with stage III ESCC also received concurrent ICI. The reason was that our institution has been involved in the ESCORT-CRT study since 2020, and we observed the acceptable toxicity in enrolled patients. Owing to the significant therapeutic effect of ICI in stage IV patients, some stage III patients wanted to receive standard CRT combined with ICI. Therefore, ICI was applied, and all patients signed the consents for this concurrent treatment. This retrospective study also serves to support the potentially practice-changing of the ongoing KEYNOTE-975, ESCORT-CRT, and RATIONALE-311 studies in confirming the pulmonary safety of definitive chemoradiotherapy combined with ICIs. It is worth noting that two published studies have explored concurrent radiotherapy/chemoradiotherapy plus camrelizumab as first-line treatment for locally advanced ESCC [27, 28], the incidence of pneumonitis was 11–15%, among which the incidence of grade 3 pneumonitis was 0–5%, and no grade 4–5 pneumonitis occurred, However, it was still limited to the small sample size (19 and 20, respectively) of phase Ib studies. It has been reported that the incidence of grade 2 radiation pneumonitis after definitive radiotherapy in esophageal cancer is 9.6–20.3%, and the incidence of grade 3 is 1.8–7.4% [29–31]. As compared to definitive radiotherapy, the addition of immunotherapy seems to maintain similar pulmonary toxicity rates. On the other hand, Zhang, et al. [32] reported that the application of anti-PD1 drugs before or during thoracic radiotherapy increased the incidence of radiation pneumonitis (27/45 vs 14/50, 60% vs 28%, *P* = 0.01). In fact, it is unclear whether there are toxicity differences between concurrent and sequential administration of RT/CRT in combination with ICIS in ESCC patients. More clinical trials and real-world data may be needed to confirm the concept. Our study showed that anti-PD‑1 mAbs administered concurrently with radiotherapy was associated with the incidence of grade ≥ 2 TRP. Although the incidence of grade 1–2 pneumonitis was considerably higher than existing data, the patients in our study received a prescribed PTV dose of 50.4–66 Gy, whereas the prescription PTV dose adopted by Zhang, et al. was 54 Gy [27, 28]. Overall, the pulmonary toxicity in patients with concurrent RT/CRT plus anti-PD‑1 mAbs in our study was tolerable, however, in clinical practice, close toxicity monitoring and follow-up should be conducted according to patients’ age, lung dose, lung function and the history of chronic lung disease, etc. in order to achieve longer survival benefits and better quality of life.

In the HOPE- 005 study [18], 41% (52/191) of patients with pneumonitis received corticosteroids, of whom 22 received immune rechallenge, and only 6 patients relapsed with grade 1–2 pneumonitis. Although the largest cohort reported that pneumonitis was associated with a higher recurrence rate (27.7%) than other types of irAEs [33], no severe recurrence of pneumonitis was observed in the HOPE-005 study. In our study, five patients underwent immune rechallenge, and only 1 patient developed grade 3 pneumonitis. Considering that treatment-related AEs of any grade were associated with higher ORR [34], and continuous application of ICIs for more than 1 year may improve clinical outcomes [35], immune rechallenge may be a viable treatment option.

DVH parameters (e.g., MLD and V_20_) can predict symptomatic radiation pneumonitis in patients with esophageal cancer who receive definitive chemoradiotherapy. The combination of ICIs and thoracic radiotherapy may amplify the risk of pulmonary toxicity. Therefore, it is more urgent to formulate constraints based on dosimetric prediction factors in the era of ICIs. There is still a lack of correlation analyses between pneumonitis and DVH parameters in patients with ESCC treated with thoracic radiotherapy combined with ICIs. In the analysis of radiotherapy combined with immunotherapy for NSCLC, conclusions regarding the relationship between DVH parameters and TRP are inconsistent, and no specific threshold for DVH parameters is recommended. Several studies have reported that V_20_ and MLD are associated with TRP [17, 18, 36]. However, another retrospective analysis from Japan showed that the incidence and severity of pneumonitis in NSCLC patients receiving concurrent chemoradiotherapy followed by durvalumab consolidation therapy were not related to total lung V_20_ [37]. Similarly, Lu et al. [38] reported that V_5_, V_20_ and MLD were not associated with the occurrence of TRP in 196 patients who received thoracic radiotherapy prior to ICIs from Chinese lung cancer cohort. Because of the rare and controversial results of related studies, it is important to identify predictors of pneumonitis after combined therapy to provide reliable data references. To our knowledge, our study is the first to define dosimetric relationships between lung dose-volume parameters and TRP after thoracic radiotherapy combined with ICIs in Asian patients with ESCC. A major finding herein was that V_5_, V_10_, V_15_, V_25_, V_30_, V_35_, V_40_, and MLD were associated with grade ≥ 2 TRP and V_5_ was an independent risk factor. This implies that V_5_ is a noteworthy predictor in addition to high-dose region, which is inconsistent with the general lack of validation in the non-ICI setting. One of the hallmarks of IMRT is its ability to improve consistency in the intermediate- and high-dose region by spreading the low dose-irradiated volume, thereby increasing the lung V_5_ [39]. IMRT was associated with a better lung toxicity profile compared to 3D-CRT [40]. The larger low dose-irradiated area of IMRT did not appear to cause more severe lung injury. But the findings of this study indicate that the intensive low dose-irradiated volume appears to be more associated with TRP after adding ICIs. That novel finding maybe due to the fact that ICIs plus RT could activate the immune system, so that even a “low dose bath” could cause pneumonitis. In this study, all patients received IMRT. Therefore, both intermediate- and high-dose region and low dose region should be treated with caution under the premise of the combination of radioimmunotherapy. The overall lung toxicity will be determined by a tradeoff between these two trends. Through the aforementioned caveat, extra care should be taken to limit V_5_ to less than 55% in IMRT planning to reduce the incidence and severity of TRP.

There were some limitations in our study. First, this was a retrospective study which might have distorted the results due to unmeasured confounding factors, selection bias and heterogeneous populations. In this analysis, patients were enrolled in single institution, and the sample size was relatively finite. Second, this study did not include the population who received thoracic radiotherapy after prior ICIs due to the heterogeneities of patients as well as the small sample size precluding further stratification analysis. This remains an issue requiring further investigation. Third, although we found that V_5_ was an independent risk factor for G2+TRP, the AUC value of 0.722 implies medium predictive value, which may also be limited by the small sample size. Further analysis with an enlarge sample size is needed to confirm our observations. Fourth, the literature reported that the median onset time of ICI-related pneumonitis was 19.2 months [23], while the median follow-up time in our study was 12.3 months, which should be remedied by a longer follow-up. Therefore, it is possible that this study still underestimates the occurrence of overall TRP, along with the fact that death is a competing risk factor for TRP in the population with poor prognosis. Fifth, there was insufficient data in this study for a robust statistical assessment of immune rechallenge. Thus, the outcome of the immune rechallenge must be interpreted with caution.

## Conclusion

In conclusion, the combination of thoracic radiotherapy/chemoradiotherapy and anti-PD‑1 mAbs displayed a tolerable pulmonary safety profile. Anti-PD‑1 mAbs administered concurrently with radiotherapy, V_5_, V_10_, V_15_, V_25_, V_30_, V_35_, V_40_ and MLD, were associated with the occurrence of grade ≥ 2 TRP. It seems safer to control V_5_ below 55% in clinical, especially for the high-risk populations receiving concurrent therapy. Due to the limited data on pulmonary toxicity in advanced ESCC patients after thoracic irradiation combined with anti-PD-1mAbs, our conclusion still needs to be confirmed by further studies.

## Supplementary Information

Supplement Figure 1

Supplement Table 1

## Data Availability

All data generated or analyzed during this study are included in this published article and its supplementary information files.

## Abbreviations

CTCAE V5.0: Common Terminology Criteria for Adverse Events Version 5.0
DVH: dose-volume histogram
ECOG: Eastern Cooperative Oncology Group
ESCC: esophageal squamous cell carcinoma
ICIs: immune checkpoint inhibitors
mAbs: monoclonal antibodies
MLD: mean lung dose
NSCLC: non-small cell lung cancer
PTV: planning target volume
ROC: receiver operating characteristic
TRP: Treatment-related pneumonitis
V_5_–V_50_: the percentage of lung volume receiving more than 5–50 Gy in increments of 5 Gy
