# Supplementary material for: Treatment-related pneumonitis after thoracic radiotherapy/chemoradiotherapy combined with anti-PD-1 monoclonal antibodies in advanced esophageal squamous cell carcinoma
Source: Strahlenther Onkol. 2024 Jan 24;200(10):857–66. doi: 10.1007/s00066-024-02199-6 (PMC11442583; doi:10.1007/s00066-024-02199-6)
Supplement: Supplementary file 1 — Supplement Figure 1 [file 66_2024_2199_MOESM1_ESM.pdf]

Baseline | **At diagnose**

Patient 1

Patient 2

Patient 3

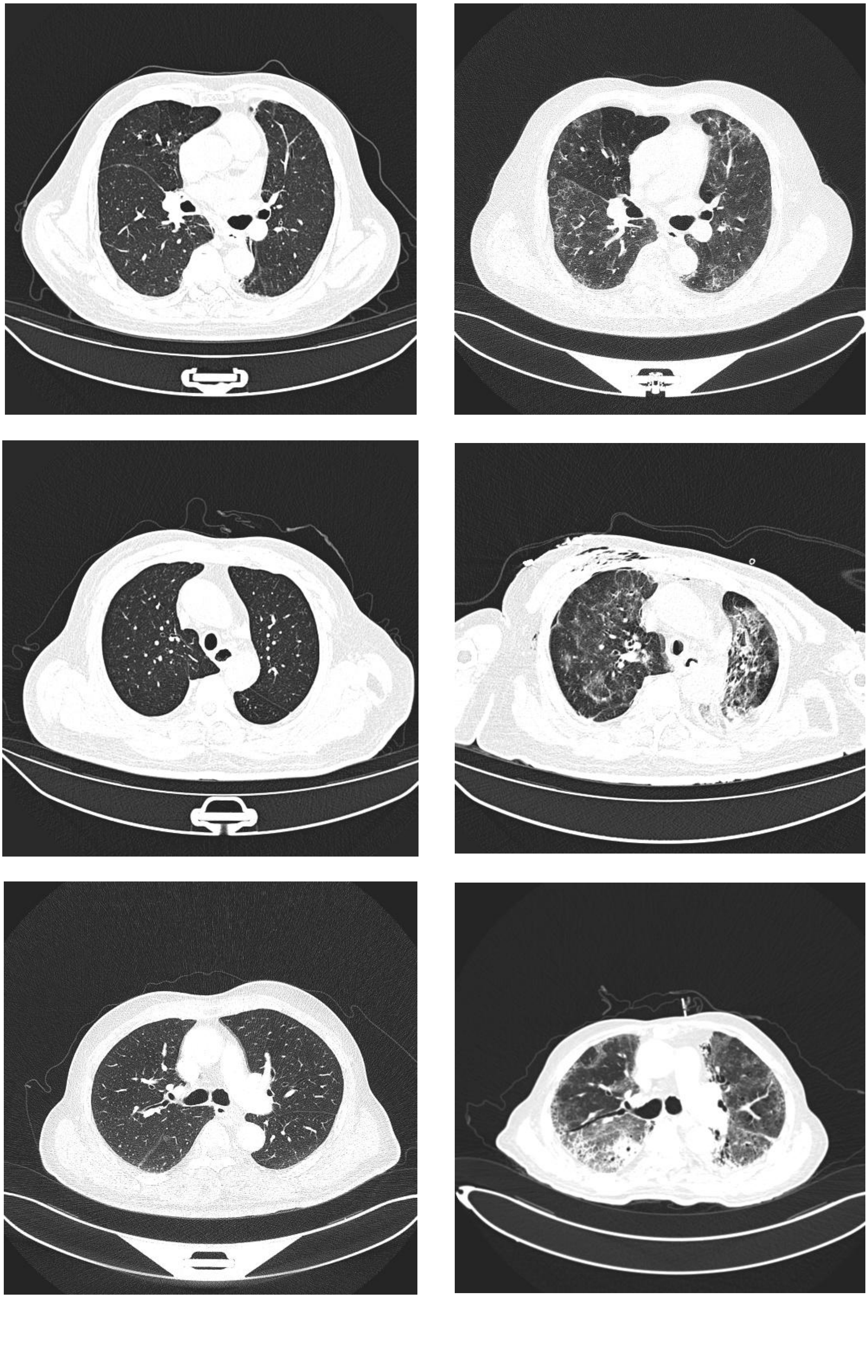

Patient 4

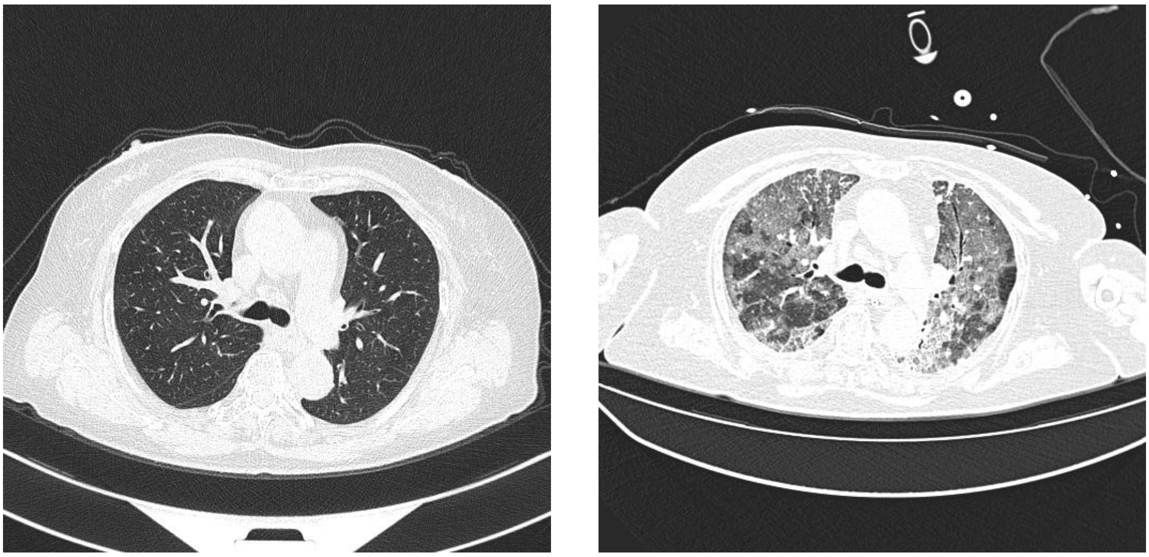

Patient 5

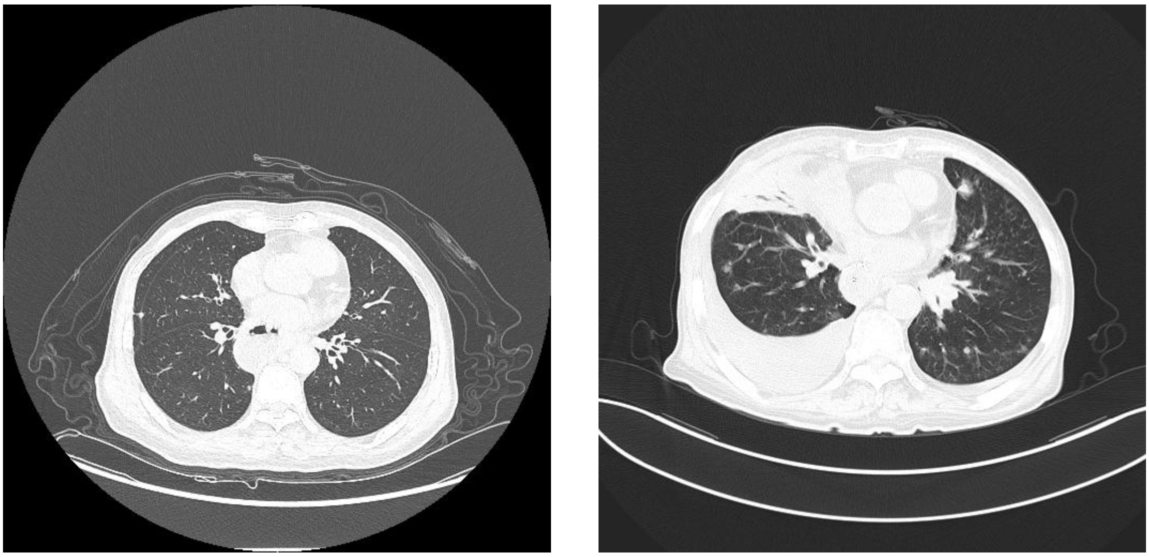

Supplement Figure 1. The CT images of 5 patients with grade 3 or higher TRP.
