# Supplementary material for: Treatment-related pneumonitis after thoracic radiotherapy/chemoradiotherapy combined with anti-PD-1 monoclonal antibodies in advanced esophageal squamous cell carcinoma
Source: Strahlenther Onkol. 2024 Jan 24;200(10):857–66. doi: 10.1007/s00066-024-02199-6 (PMC11442583; doi:10.1007/s00066-024-02199-6)
Supplement: Supplementary file 2 — Supplement Table 1 [file 66_2024_2199_MOESM2_ESM.pdf]

Supplement Tab. 1. Individual characteristics and treatment of 5 patients with G3+ pneumonitis.

Characteristics	Patient 1	Patient 2	Patient 3	Patient 4	Patient 5
Grade of TRP	3	3	3	3	5
Demographics					
Age	65	74	68	78	76
Sex	Male	Male	Male	Male	Female
Smoking history	Yes	Yes	Yes	Yes	No
Pulmonary disease	None	Emphysema	None	None	None
Modality					
Concurrent chemotherapy	TP	TP	TP	T	Tegafur
RT combined with ICI	Sequential	Concurrent	Concurrent	Concurrent	Sequential
Dosimetry					
V_5(%)	55.2	58.6	47.6	59.8	59.5
V_{10}(%)	40.5	40.9	34.8	39.7	43.9
V_{20} (%)	23.9	29.1	20.1	21.3	24.1
V_{30}(%)	14.9	16.6	13.0	12.2	13.3
MLD(Gy)	1321.3	1387.9	1063.2	1224.5	1307.6
Occurrence of TRP					
The intervals between the initiation of RT and TRP (days)	516	51	40	110	153
The intervals between the initiation of ICIs and TRP (days)	427	63	47	117	59
Treatment					
Corticosteroid	Yes	Yes	Yes	Yes	Yes
Mechanical ventilation support	No	No	No	No	Yes

Abbreviations: TP, Paclitaxel and cisplatin; T, Paclitaxel; V_5, percentage of lung volume receiving more than 5 Gy; V_{10}, percentage of lung volume receiving more than 10 Gy; V_{20}, percentage of lung volume receiving more than 20 Gy; V_{30}, percentage of lung volume receiving more than 30 Gy; MLD, mean lung dose.
